# Validity of Antibodies in Lymphocyte Supernatant in Diagnosing Tuberculosis in Severely Malnourished Children Presenting with Pneumonia

**DOI:** 10.1371/journal.pone.0126863

**Published:** 2015-05-28

**Authors:** Mohammod Jobayer Chisti, Mohammed Abdus Salam, Rubhana Raqib, Sayera Banu, Abu ASMSB Shahid, KM Shahunja, Lazina Sharmin, Hasan Ashraf, Abu Syed Golam Faruque, Pradip Kumar Bardhan, Tahmeed Ahmed

**Affiliations:** 1 International Centre for Diarrhoeal Disease Research (icddr,b), Bangladesh, Dhaka, Bangladesh; 2 Centre for International Child Health, The University of Melbourne Department of Paediatrics and Murdoch Children’s Research Institute, Royal Children’s Hospital, Melbourne, Australia; University of Southampton Faculty of Medicine, UNITED KINGDOM

## Abstract

**Background:**

The diagnosis of tuberculosis (TB) in young children can be challenging, especially in severely malnourished children. There is a critical need for improved diagnostics for children. Thus, we sought to evaluate the performance of a technique that measures antibodies in lymphocyte supernatant (ALS) for the diagnosis of TB in severely malnourished children presenting with suspected pneumonia.

**Methods:**

Children less than 5 years with severe acute malnutrition and radiological features of pneumonia admitted to the Dhaka Hospital of International Centre for Diarrhoeal Disease Research, Bangladesh, were enrolled consecutively following informed written consent. In addition to clinical and radiological assessment, samples taken for TB diagnosis included gastric lavage fluid and induced sputum for microbiological confirmation. ALS was measured from venous blood, and results were evaluated in children classified as “confirmed”, “non-confirmed TB” or “not TB”.

**Results:**

Among 224 children who had ALS analysis, 12 (5.4%) children had microbiologically “confirmed TB”, a further 41 (18%) had clinically diagnosed “non-confirmed TB” and the remaining 168 (75%) were considered not to have TB. ALS was positive in 89 (40%) and negative in 85 (39%) of children, with a large number (47 or 21%) reported as “borderline”. These proportions were similar between the three diagnostic groups. The sensitivity and specificity of ALS when comparing “Confirmed TB” to “Not TB” was only 67% (95% CI: 31–91%) and 51% (95% CI: 42–60%), respectively.

**Conclusions and Significance:**

Our data suggest that ALS is not sufficiently accurate to improve the diagnosis of TB in children with severe malnutrition.

## Introduction

The 2013 global tuberculosis report of the World Health Organization (WHO), based on vital registration data, estimated that TB caused 74,000 deaths in HIV-uninfected children globally in 2012 [1]. Recent data suggest that TB in under-15 children may contribute 10–20% of the total disease burden in endemic countries [2,3]. Due to lack of reporting of TB as a cause of deaths in HIV and/or pneumonia related deaths [1,4,5], the actual burden of childhood TB is likely to be higher than these estimates. However, the main impediment to understand the actual burden is the difficulties in the confirmation of diagnosis of TB in children. Obtaining sputum samples from young children can be challenging and disease is paucibacillary, so yield from microscopy is low. Mycobacterial culture takes as long as 8–12 weeks for a result in conventionally used solid cultures, and while real-time PCR technique such as Xpert MTB/RIF assay is much quicker, sensitivity is suboptimal compared to culture [6], so a negative result does not rule out a diagnosis of TB. Undoubtedly, there is a huge need for an accurate and rapid diagnostic test for TB in children.

Our group has previously published novel data of encouraging results from evaluating a blood-based test measuring antibodies in lymphocyte supernatant (ALS) for diagnosis of TB in adults and children [7,8]. The study in children was limited to those with a clinical diagnosis of TB. Among 58 children with clinically diagnosed TB, 9 (15%) had culture-confirmed and 53 (91%) had ALS positive TB [8]. Further, the levels of ALS may be affected by reduced immune function such as occurs in children with severe malnutrition. We recently conducted a prospective study of the prevalence of TB among children with severe malnutrition and radiologic pneumonia [9]. In a subset of children from that study, we have evaluated the diagnostic performance of ALS in the diagnosis of TB in comparison with culture and Xpert MTB/RIF in addition to comparison with clinical diagnoses of childhood TB.

## Materials and Methods

### Ethics statement

The study was approved by the Research Review Committee (RRC) and the Ethical Review Committee (ERC) of the International Centre for Diarrhoeal Disease Research, Bangladesh (icddr,b). Institutional Review Board of icddr,b comprises of RRC and ERC. Written informed consent was obtained from parents or guardians of each of the participating children; children whose caregivers did not give consent were not enrolled.

### Study design

Details of the study population, study setting and clinical management have been comprehensively described previously [9]. Briefly, consecutive young children (< 5 years) with severe malnutrition and respiratory symptoms (cough and/or respiratory distress) with radiological evidence of pneumonia were enrolled following informed consent in a prospective cohort study conducted at the Dhaka Hospital of icddr,b between April 2011 and June 2012 [9]. Detailed clinical, epidemiological and demographic data were collected in addition to gastric lavage fluid and induced sputum for microscopy for acid-fast bacilli and mycobacterial culture, as well as for real-time PCR to identify *Mycobacterium tuberculosis* by the Xpert MTB/RIF assay once it became available during the study. All children were classified on the basis of study definitions as having either “confirmed TB”, “non-confirmed” TB or “not TB” [9].

Severe malnutrition was defined as the presence of severe wasting [Z score for weight for height <-3 of the WHO median] or severe under-weight [Z score for weight for age <-4 of the WHO median], or nutritional edema. “Confirmed TB” was defined as the identification of *M tuberculosis* by culture or by Xpert MTB/RIF assay on any of the test specimens. “Non-confirmed TB” was diagnosed clinically with supportive evidence such as positive tuberculin skin test (TST) or a positive contact history or when there was no symptomatic improvement of bacterial pneumonia or severe malnutrition following therapy (without microbiological confirmation of TB). “Not TB” included all other children who were enrolled in the study and had completed the assessments (i.e. sputum sample collected and TST performed and interpreted) but did not fulfill the criteria of either “Confirmed TB” or “Non-confirmed TB”.

In a sub-set of children from that study, we sampled blood for measurement of ALS. Laboratory procedure for ALS has been described earlier. [8]. Briefly, the ALS assay measures spontaneous release of TB-antigen-specific IgG antibodies from *in vivo*-activated plasmablasts using *ex vivo* cultures of unstimulated peripheral blood mononuclear cells (PBMC) followed by an ELISA [8,10]. In brief, PBMC were isolated from 2.5 to 3 ml of blood by using Ficol-Paque density gradient centrifugation (GE Healthcare Bio Science AB, Uppsala, Sweden) and cultured in (5x10^6^ cells/ml in 48-well plates; Thermo Fischer Scientific, NUNC, Roskilde, Denmark) in RPMI medium (Gibco, Life technologies, Green Island, NY) for 48 h. Release of IgG antibodies in the culture supernatant was measured using BCG-specific ELISA. The BCG vaccine was obtained from Japan BCG Laboratories, Tokyo, Japan. The ALS response is expressed as relative BCG-specific IgG titers or optical density (OD); ALS results were reported as “positive” if antibody titer was ≥ 0.60, “negative” if antibody titer was < 0.38, or “borderline” if antibody titer was ranged from 0.38 to 0.59. In addition we assessed the performance of ALS at different cut-offs as follows: OD ≥ 0.42 as “positive” and OD < 0.42 as “negative”; and OD ≥ 0.35 as “positive” and OD < 0.35 as “negative”.

### Analysis

All data were entered onto a computer using SPSS for Windows (version 17.0; SPSS Inc, Chicago) and Epi-Info (version 6.0, USD, Stone Mountain, GA). We determined the proportion of children that had positive, negative or borderline ALS results within each of the TB categories. The differences in proportions were compared by the Chi-square test. The differences in normally distributed continuous data were compared by Student’s t-test and Mann-Whitney test was used for comparison of data that were not normally distributed. A probability of less than 0.05 was considered statistically significant. The strength of association was determined by calculating odds ratio (OR) and their 95% confidence intervals (CIs). We calculated the sensitivity, specificity, positive and negative predictive values of ALS at different cut-offs for the diagnosis of TB in severely malnourished children by comparing results between those with “Confirmed TB” and those classified as “Not TB”, and by comparing all TB cases (“Confirmed TB” plus “Non-confirmed TB”) to “Not TB”.

## Results

Among the 405 children enrolled during the study period [8], ALS from a blood sample was measured in the first 224 children. Of these, 221 children had gastric lavage fluid and induced sputum obtained for microscopy and mycobacterial culture, and 51 of these children also had gastric lavage fluid and induced sputum analyzed by Xpert MTB/RIF. Only one sample of gastric lavage fluid and induced sputum was taken from each study patient and microscopy, mycobacterial culture, and Xpert MTB/RIF were done both from the gastric lavage fluid and induced sputum of each patient. The median (IQR) age of the study children was 10 (5, 16) months and age range was 1 to 59 months. Of the 221 study children, there were 12 (5.4%) children with microbiologically confirmed TB—8 by culture and 5 by X-pert MTB/RIF (1 positive in both tests)—and there were 41 (19%) with clinical, non-confirmed TB and 168 that were considered not to have TB.

Overall, ALS was positive in 89/221(40%), negative in 85/221 (39%), and borderline in 47/221 (21%) children, and the proportion that were positive, negative or borderline are listed in Table 1. The proportion of positive ALS was found to have no significant difference among study children who had “Confirmed TB”, “Non-confirmed TB”, and “Not TB” (Chi-Square for linear trend for positive ALS from “Confirmed TB” to “Non-confirmed TB” to “Not TB” = 1.098, p = 0.295). The clinical characteristics of the severely malnourished children with pneumonia are compared between those with and without a positive ALS in Table 2. The characteristics of the group of children with a positive ALS result were similar to those of the group of children with a negative ALS result, although the former were significantly older on average, and a significantly greater proportion was BCG-vaccinated. The sensitivity, specificity, positive predictive value, negative predictive value and accuracy of ALS at different cut-offs for “confirmed TB” versus “not TB”; and for “all TB” versus “not TB” are shown in Table 3. Although, the sensitivity of ALS with the lower cut-offs was increased from 67% to 75% for “confirmed TB” versus “not TB” and from 57% to 66% for “all TB” versus “not TB”, however, the specificity and accuracy of ALS with the lower cut-offs gradually decreased from 51% to 39% and 52% to 41% for “confirmed TB” versus “not TB” and 51% to 39% and 52% to 45% for “all TB” versus “not TB—respectively. Baseline characteristics of severely malnourished children according to diagnostic groups (confirmed TB”, “Non-confirmed TB”, and “Not TB”) are shown in Table 4.

**Table 1:** Summary results of ALS in blood (using an optical density ≥ 0.60 as a positive cut-off and < 0.38 as a negative cut-off), confirmed TB, non-confirmed TB, and not TB in children under five with suspected pneumonia and severe malnutrition.

Parameters	Number of patients	ALS positive	ALS borderline	ALS negative
Confirmed TB	12	6 (50%)	3 (25%)	3 (25%)
Non-confirmed	41	19 (46%)	6 (15%)	16 (39%)
Not TB	168	64 (38%)	38 (23%)	66 (39%)
Total	221	89 (40%)	47 (21%)	85 (39%)

**Table 2:** Characteristics of severely malnourished children with suspected pneumonia who had their ALS done.

Variables	ALS positive	ALS negative	p
	n = 89	n = 85	
	n (%)	n (%)	
Age (months) (median, IQR)	12 (6.8, 18.7)	8.0 (3.5, 15.0)	0.002
Male	52 (58)	51 (56)	0.898
History of contact with sputum positive TB	5 (6)	3 (4)	0.721
Poor socio-economic condition*	73 (80)	78 (91)	0.108
BCG vaccination	83 (93)	66 (78)	0.007
Tuberculin skin test positive	18 (20)	17 (20)	0.879
Z score (W/A) (mean ± SD)	-4.8±1.3	-4.9±1.3	0.430
Z score (W/L) (mean ± SD)	-4.1±1.3	-3.6±1.5	0.041
Cough	84 (93)	79 (92)	0.931
Respiratory rate	49 ± 12	51 ± 12	0.319
Hypoxemia (SPO2 < 90% at sea level)	6 (7)	8 (9)	0.713
Bacterial isolates in blood	5 (6)	4 (5)	1.00
Confirmed TB	6 (7)	3 (4)	0.279
Non-confirmed TB	19 (21)	16 (19)	0.881
Not TB	64 (71)	66 (77)	0.881

IQR = Inter-quartile range; SD = Standard deviation

*monthly income <USD 125

**Table 3:** Validity of ALS at different cut-offs for “Confirmed” versus “Not TB”; and for “all TB” (“Confirmed” plus “Non-Confirmed”) versus “Not TB”.

ALS cut-off (OD)	Comparison of ALS validity for “confirmed” versus “not TB”					Comparison of ALS validity for “all TB” versus “not TB”				
	Sensitivity	Specificity	PPV	NPV	Accuracy	Sensitivity	Specificity	PPV	NPV	Accuracy
	(95% CI)	(95% CI)	(95% CI)	(95% CI)		(95% CI)	(95% CI)	(95% CI)	(95% CI)	
	(%)	(%)	(%)	(%)	(%)	(%)	(%)	(%)	(%)	(%)
≥0.60	67 (31–91)	51 (42–60)	9 (4–18)	96 (87–99)	52%	57 (41–71)	51 (42–60)	28 (19–39)	78 (67–86)	52%
≥0.42	67 (35–89)	45 (37–52)	8 (4–15)	95 (87–98)	46%	60 (46–73)	45 (37–52	26 (18–34)	78 (68–86)	48%
≥0.35	75 (43–93)	39 (31–47)	8 (4–15)	96 (87–99)	41%	66 (52–78)	39 (31–47)	25 (19–34)	78 (68–86)	45%

OD = Optical density; CI = Confidence intervals; PPV = Positive predictive value; NPV = negative predictive value; All TB = “confirmed TB” and “Non-confirmed TB”

**Table 4:** Baseline characteristics of study participants according to diagnostic groups.

Variables	Confirmed TB (n = 12)	Non-confirmed TB (n = 41)	Not-TB (n = 168)
Age (months) (median, IQR)	13.5 (2.7, 24.0)	11.0 (5.8, 16.5)	10.0 (4.9, 15.0)
Male	9 (75)	24 (59)	91 (54)
History of contact with sputum positive TB	3 (25)	4 (10)	2 (1)
BCG vaccination	10 (83)	38 (93)	138 (82)
Poor socio-economic condition	11 (92)	34 (83)	145 (86)
Z score (W/A) (mean ± SD)	-5.5±0.9	-4.9±1.4	-5.0±1.4
Z score (W/L) (mean ± SD)	-4.2±2.0	-4.2±1.3	-3.8±1.6
Cough	11 (92)	38 (93)	156 (93)
Duration of cough (days) (median, IQR)	7.0 (4.0, 15.0)	5.0 (4.0, 7.0)	5.0 (3.0, 7.0)
Fever	8 (67)	30 (73)	124 (74)
Duration of fever (days) (median, IQR)	15.0 (7.0, 30.0)	5.0 (3.0, 8.0)	4.0 (3.0, 6.8)
Poor feeding	1 (8)	7 (17)	17 (10)
Duration of poor feeding (days) (median, IQR)	10 (10.0, 10.0)	15.0 (7.0, 30.0)	4.0 (2.0, 10.0)
Respiratory rate	47 ± 14	49 ± 12	50 ± 11
Bacterial isolates in blood	0	2 (5)	8 (5)
Tuberculin skin test positive	1 (8)	39 (95)	0

## Discussion

The important observation of this study is the poor performance of ALS in the diagnosis of TB in our study population. This test was originally validated in an adult population and found to have a sensitivity and specificity of 92.5% and 80% compared to smear positive and microbiologically confirmed TB, respectively [7]. Also, in a previous study investigating ALS in children with clinically diagnosed TB (age range: 11 to 167 months) the sensitivity and specificity of the test were 91% and 87%, respectively [8]. However, in this population of severely malnourished and young children, the diagnostic accuracy of the ALS was poor including in children with microbiologically confirmed TB.

The ALS test is based on the assumption that antigen-specific plasma cells are present in the circulation only at times of active TB disease and not in asymptomatic infections i.e. in latent TB infection [8]. ALS is thus expected to have greater specificity for active disease because it quantifies the amount of antibody actively secreted by TB-specific plasma cells [11]. Based on this, ALS could be expected to perform well in cases with microbiologically confirmed TB. However, among the study children who were considered not to have TB (i.e. who did not have either confirmed or non-confirmed TB), 38% of those children were observed to have a positive ALS result, which either indicates that there are significant issues with test specificity or alternatively could indicate that the ALS assay also produces positive results in children with latent TB infection who coincidentally have another respiratory infection. Notably, none of the children in the “not TB” group had a positive TST result, but it is well-documented that false-negative TST results are common in children with severe malnutrition [12,13]. Due to financial constraints we were not able to perform interferon-gamma release assays (IGRAs), which could have further contributed to excluding potential latent TB infection in those children. One possible explanation for the poor performance of ALS in our study population is that both cell mediated and humoral (i.e. plasma cell function) immune responses are substantially impaired in children with severe malnutrition [14,15]. It is worth noting that our study participants were on average considerably younger than the participants of the two previous studies that evaluated ALS. Similar to other tests that rely on immune response, it is possible the immaturity of the immune system in our study participants has resulted in an increase in the proportions of negative or borderline results, a phenomenon also observed with IGRAs [16,17,18].

We have considered the performance of ALS at different cut-offs. Although, at lower cut-offs the sensitivity of ALS improved slightly, simultaneously false positivity of ALS was increased, thus, resulting in reduction of the specificity and accuracy of ALS that potentially make the performance of the test worse.

The main limitation of the study is a small number of children with microbiologically-confirmed TB that may hinder the actual ability to assess test accuracy. The vast majority of cases were classified as ‘non-confirmed’ TB, some of whom might not have had TB. This may lead to data contamination, which might make test performance appear worse than it actually is. However, our approach to assess the performance of the ALS by comparing children with ‘only confirmed TB’ versus ‘not TB’ intended to address this limitation.

In conclusion, measurement of ALS does not appear to have a role in the diagnosis of TB in severely malnourished children in our population.
